# Shoot-soil ecological stoichiometry of alfalfa under nitrogen and phosphorus fertilization in the Loess Plateau

**DOI:** 10.1038/s41598-021-94472-2

**Published:** 2021-07-22

**Authors:** Jiaoyun Lu, Hong Tian, Heshan Zhang, Junbo Xiong, Huimin Yang, Yang Liu

**Affiliations:** 1 Key Laboratory of Animal Embryo Engineering and Molecular Breeding of Hubei Province, Institute of Animal Husbandry and Veterinary, Hubei Academy of Agricultural Science, Yaoyuan 1, Hongshan, Wuhan, 430064 China; 2 State Key Laboratory of Grassland Agro-ecosystems, College of Pastoral Agriculture Science and Technology, Lanzhou University, 768 Jiayuguanxi Road, Lanzhou, 730020 China

**Keywords:** Ecology, Physiology, Plant sciences

## Abstract

Plants and soil interactions greatly affect ecosystems processes and properties. Ecological stoichiometry is an effective means to explore the C, N, P correlation between plants and soil and the relationship between plant growth and nutrient supply. Serious soil erosion on China’s Loess Plateau has further barrenness the soil. Fertilization solves the problem of ecosystem degradation by improving soil fertility and regulating the ecological stoichiometric between soil and plants. No fertilization (CK), nitrogen fertilization (N), phosphorus fertilization (P) and N and P combined fertilization (NP) treatments were set in an alfalfa grassland. Organic carbon (C), nitrogen (N) and phosphorus (P) nutrients and their stoichiometry were measured in shoot and soil. P and NP fertilization increased shoot C concentration (3.12%, 0.91%), and all fertilization decreased shoot N concentration (6.96%). The variation of shoot C and N concentrations resulted in a greater increase in shoot C:N under the fertilization treatment than that under CK (8.24%). Most fertilization treatments increased shoot P concentration (4.63%) at each cut, which induced a decrease of shoot C:P. Shoot N:P of most treatments were greater than 23, but it was lower under N and NP fertilization than that under CK. Fertilization only increased soil C in 2014, but had no effect on soil N. Soil P content was significantly higher under P fertilization in 2014 (34.53%), and all fertilization in the second cut of 2015 (124.32%). Shoot and soil C:P and N:P having the opposite changes to shoot and soil P, respectively. Our results suggest that the change of P after fertilization largely drove the changes of stoichiometric. The growth of alfalfa in the Loess Plateau was severely restricted by P. It is an effective method to increase the biomass of alfalfa by increasing the addition of N or NP fertilizer to alleviate P limitation.

## Introduction

China’s Loess Plateau is the world’s largest area of eolian loess deposits and has restricted availability of soil nutrients^1^. A series of measures focusing on soil conservation have been carried out in this region. Maintaining and improving soil fertility are two of the main targets of farming and animal husbandry production and management^2^. Fertilization is an important measure for improving soil fertility; it can influence soil nutrient status and the growth of the plant, then promote the productivity of the system^3,4^. Nitrogen (N) and phosphorus (P) fertilizers, either individually or in combination, improve primary productivity in most terrestrial ecosystems^5^. These fertilizers can change the proportion of the N and P structure in the soil, regulate the growth strategies and nutrient dynamics of plants in response to these soil nutrients, and then affect plant productivity^6,7^. N and P fertilizer application not only increases organic carbon (C), N, and P in the soil, but also improves N and P uptake by stimulating plant growth^8,9^, which is more obvious in nutrient-limited regions^10^. These studies mainly focused on the effects of N or P fertilizer interacting with other factors on plant and soil nutrients. There are few studies about the effects of combined N and P fertilization on C, N, and P nutrients in plant and soil.

C, N, and P play a key role in the process of plant growth and have been widely studied in ecological stoichiometry^11^. Ecological stoichiometry provides a new comprehensive method to study the relationship and regularity of elements in the ecological process and relationship between nutrient supply and plant growth^12,13^. The C:N:P ratio has often been used to explore the relationships and feedback between above- and below-ground components of ecosystems^14^. In ecological process, fertilization increases the nutrient supply in the soil, promotes the nutrient uptake and stimulates plant growth, thereby regulating C, N, and P balance in the soil and plants, and improves the adaptability of plants to the environment^9,15–17^. N fertilization could either increase crop C:P and N:P but decrease C:N^7,17^ or increase N:P but decrease C:N and C:P^18,19^, and P fertilization reduce C:P and N:P in terrestrial systems^19^. Further, legumes (*Caragana microphylla*) respond modestly to the changes of soil nutrient availability resulting from N addition^8^. Changes in plant growth rate lead to differences in nutrient demand, soil heterogeneity leads to differences in nutrient supply, and differences in element uptake by plants in response to different environments all affect nutrient content and distribution in the plant–soil system, which increase the complexity of the nutrient relationship within the system^19,20^. C:N:P produces unpredictable changes in plants, which suggests that plant stoichiometry may not be a simple reflection of soil nutrient availability^15^. The type of soil/plant nutrient limitation and the genetic characteristics of species also affect plant and soil stoichiometry. Therefore, according to local conditions, exploring the shoot-soil nutrient stoichiometric ratio changes of endemic species changes under fertilization (especially N and P combined), is of great significance for clarifying the rule of systematic nutrient utilization and circulation under fertilization and for guiding fertilization management.

Alfalfa (*Medicago sativa*) is a high-quality forage plant that has been extensively cultivated in China’s Loess Plateau^21^. However, infertile soil constrains the production and sustainable use of alfalfa^21,22^. Soil N and P decrease monotonically with alfalfa growth and frequent cutting; therefore, fertilization is needed in practice to balance soil nutrients and to postpone the degradation of alfalfa grassland^23^. Because alfalfa is a N_2_-fixing species, P plays a key role in its growth. However, excessive addition of P fertilizer will increase the residual P in the soil, resulting in a waste of resources^21^. Therefore, optimized N and P management is of significant importance not only for improving the sustainability of forage production and quality, but also for reducing the economic input and environmental pollution^21,24^. Therefore, it is of great significance to study the effects of N and P supply on the shoot-soil stoichiometry of alfalfa grassland, for improving the soil nutrient balance, alleviating nutrient limitation in alfalfa growth, and to improving its adaptability and productivity in this infertile and arid region.

Herein, the present study focuses on alfalfa in Qingyang, Gansu Province, a typical Loess Plateau area of China. Using field sampling and laboratory experiments, the C, N, and P stoichiometric characteristics of the shoot-soil area were studied. The purposes of this study were: (1) to clarify the C, N, and P stoichiometric characteristics of the alfalfa shoots and soil in the Loess Plateau; (2) to explore the influences of N and P fertilization on C, N, and P, and the stoichiometric characteristics of alfalfa shoots and soil; and (3) to reveal the adaptation strategy of alfalfa to the change of soil nutrient availability in nutrient barrenness environment on the Loess Plateau. The results of the present study may improve ecological stoichiometry theory, provide references for reasonable fertilization, and improve the quality of alfalfa-cultivated grassland.

## Results

### Changes of C, N, and P concentration and stoichiometry in alfalfa

P and NP fertilization increased shoot C concentration at the first cut in 2014 and 2015, which significantly increased at the 2014 (Table 1). N fertilization had no effect on C concentration. P and NP fertilization only increased shoot C concentration at the first cut of 2014. Shoot N concentration generally declined after fertilizer treatment in each cut of the three years and was significantly lower under P fertilization at the first cut of 2014. All fertilization increased shoot P concentration at each cut, except that P fertilization in 2014, N and NP fertilization in the second cut of 2015 decreased shoot P concentration. Generally, P and NP increased shoot C concentration, fertilization decreased shoot N, but increased shoot P.

**Table 1:** Characteristics of alfalfa C, N, and P concentration under N and P fertilization.

Index	Treatment	Sample time			
		2014-1st	2015-1st	2015-2nd	2016-1st
C (g kg^−1^)	CK	420.11 ± 4.18b	429.82 ± 16.01	444.71 ± 13.47ab	438.90 ± 7.09
	P	440.08 ± 16.58a	452.70 ± 6.88	464.13 ± 11.57a	430.18 ± 17.34
	N	416.27 ± 10.79b	396.50 ± 15.10	458.99 ± 14.13a	433.10 ± 18.72
	NP	443.95 ± 5.28a	431.70 ± 59.99	431.66 ± 13.18b	440.92 ± 16.11
N (g kg^−1^)	CK	30.91 ± 2.31a	26.24 ± 4.21	27.77 ± 1.09	27.97 ± 3.44
	P	28.29 ± 0.37b	23.68 ± 0.57	25.55 ± 2.68	25.09 ± 4.68
	N	29.25 ± 0.19ab	26.26 ± 0.82	26.08 ± 0.64	22.80 ± 2.53
	NP	29.71 ± 1.09ab	25.16 ± 1.49	25.30 ± 0.49	27.90 ± 4.63
P (g kg^−1^)	CK	0.91 ± 0.09b	0.84 ± 0.05b	0.93 ± 0.02ab	1.09 ± 0.16
	P	0.55 ± 0.03c	0.89 ± 0.04b	1.00 ± 0.07a	1.08 ± 0.21
	N	0.96 ± 0.05b	0.88 ± 0.04b	0.83 ± 0.04bc	1.26 ± 0.08
	NP	1.25 ± 0.04a	1.12 ± 0.02a	0.76 ± 0.15c	1.28 ± 0.20

Values are presented as mean ± SD (*n* = 3). Different lowercase letters indicate significant differences (*P* < 0.05) between different treatments, no letter indicates no significant difference between treatments. C: organic carbon; N: total nitrogen; P: total phosphorus. The same below.

Fertilization increased shoot C:N in each of the three years, which was significantly at the first cut of 2014 and the second cut of 2015 (Table 2). With the exception of the second cut in 2015, N and NP fertilizer decreased shoot C:P in all 3 years. This effect was significant for NP fertilization in 2014 and 2015. P fertilization only significantly increased C:P in the first year. Both N and NP fertilizers decreased alfalfa N:P at the first cut of each of the 3 years, and with the extension of planting, the decreasing trend was more obvious. P fertilizer significantly increased shoot N:P at the first cut of 2014, and then decreased N:P with the alfalfa growth. Generally, fertilization increased shoot C:N, N and NP fertilization decreased shoot C:P and shoot N:P.

**Table 2:** Characteristics of shoot C:N, C:P and N:P under N and P fertilization.

Index	Treatment	Sample time			
		2014-1st	2015-1st	2015-2nd	2016-1st
C:N	CK	13.65 ± 1.16b	16.75 ± 3.48	16.02 ± 0.29b	15.86 ± 2.05
	P	15.56 ± 0.60a	19.13 ± 0.70	18.30 ± 1.99a	17.65 ± 4.04
	N	14.23 ± 0.28ab	15.10 ± 0.53	17.61 ± 0.95ab	19.19 ± 2.72
	NP	14.96 ± 0.66ab	17.18 ± 2.43	17.06 ± 0.46ab	16.03 ± 2.09
C:P	CK	466.54 ± 50.38b	510.27 ± 21.04a	476.55 ± 14.65	406.75 ± 54.82
	P	807.12 ± 63.28a	510.04 ± 19.65a	463.67 ± 33.97	411.12 ± 88.41
	N	435.81 ± 18.77b	450.96 ± 33.58ab	553.79 ± 7.35	344.67 ± 27.33
	NP	355.54 ± 14.79c	385.72 ± 58.60b	581.83 ± 116.4	347.55 ± 42.58
N:P	CK	34.14 ± 0.82b	31.23 ± 5.65a	29.75 ± 0.88ab	25.66 ± 1.44a
	P	51.88 ± 3.56a	26.67 ± 0.95ab	25.40 ± 0.96b	23.49 ± 3.13ab
	N	30.64 ± 1.43b	29.83 ± 1.18a	31.52 ± 2.14ab	18.09 ± 1.55c
	NP	23.79 ± 1.00c	22.45 ± 1.10b	33.99 ± 6.03a	21.70 ± 0.36b

### Changes of C, N, and P contents and stoichiometry in the soil

**Figure 1:**
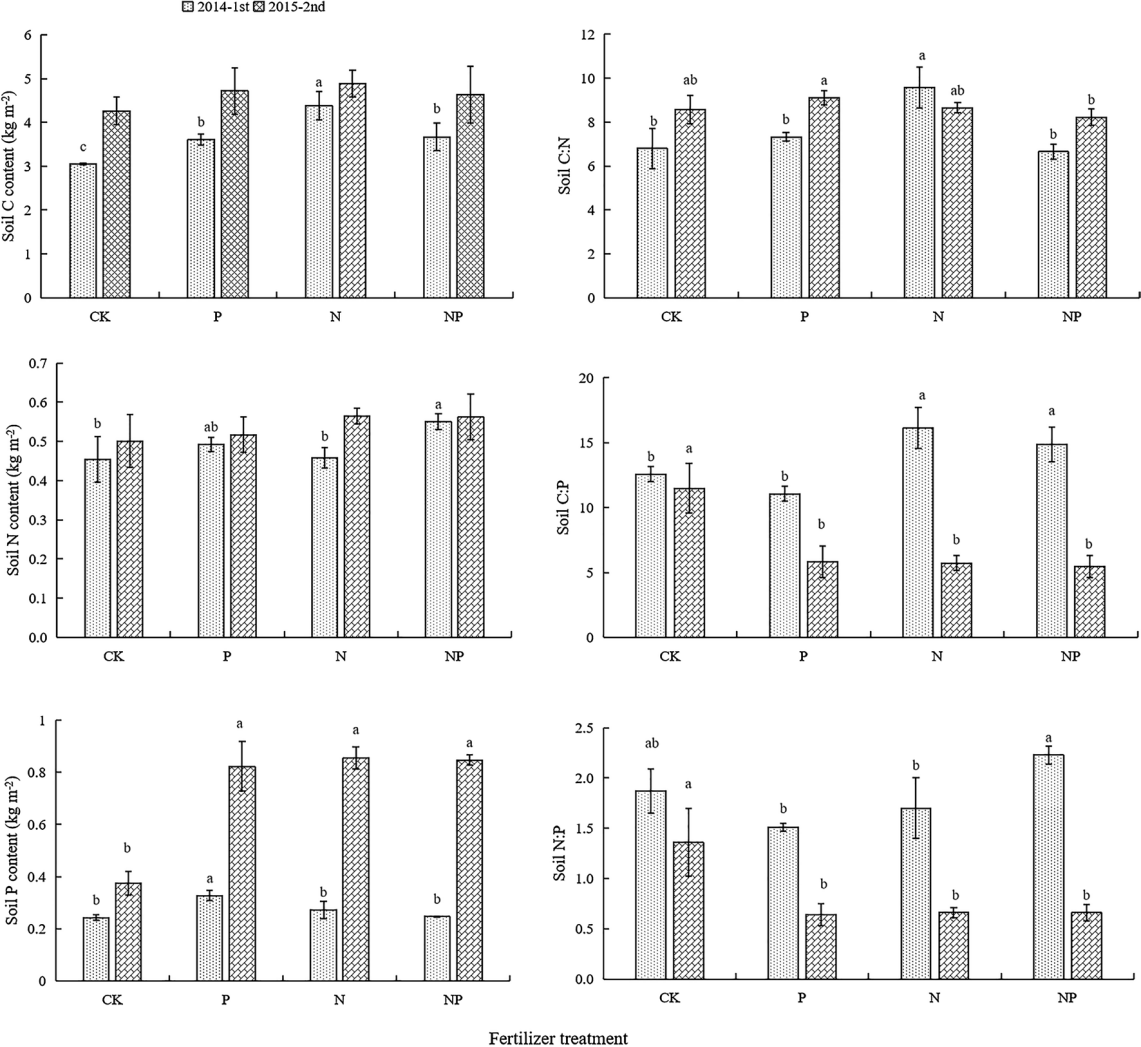
Characteristics of soil C, N, and P content and stoichiometry under N and P fertilization. Values are presented as mean ± SD. (*n* = 3). Different lowercase letters indicate significant differences (*P* < 0.05) between different fertilization treatments, no letter indicates no significant difference between different treatments. C: organic carbon; N: total nitrogen; P: total phosphorus.

Fertilization increased soil C content in both 2014 and 2015, though this was significant only in 2014 (Fig. 1). Soil N content was higher under fertilization treatment than CK, and this effect was significant under NP fertilization in 2014. Only P fertilization increased soil P in 2014, but all fertilization treatments increased soil P in 2015. Generally, fertilization increased soil C and soil P, and soil N respond modestly to the fertilization.

N fertilization significantly increased soil C:N at 2014, but fertilization had no effect on soil C:N in 2015 (Fig. 1). N and NP fertilization increased soil C:P in 2014; however, all fertilization decreased soil C:P in 2015. Fertilization had no effect on soil N:P in 2014, but soil N:P was significantly higher under NP fertilization than under N or P fertilization. Soil N:P in 2015 was significantly lower under fertilization treatments than CK. Generally, most fertilization decreased soil C:P and soil N:P.

### Changes of the available nutrients in soil

P and NP fertilizer reduced soil NN in 2014, but N and NP fertilizer increased soil NN in the other years (Table 3). In 2014, P and NP fertilizer increased soil AN, but N fertilizer decreased it. Fertilization had no effect on soil AN at the two cuts in 2015. In 2016, soil AN increased under N and NP fertilization. Fertilization increased soil AP at all cuts.

**Table 3:** Characteristics of soil nitrate nitrogen (NN), ammonium nitrogen (AN) and available phosphorus (AP) contents under N and P fertilization.

Index	Treatment	Sample time			
		2014-1st	2015-1st	2015-2nd	2016-1st
NN (g m^−2^)	CK	26.70 ± 2.38a	1.46 ± 0.46c	1.49 ± 0.50b	0.70 ± 0.22c
	P	5.08 ± 0.29c	1.41 ± 0.31c	1.71 ± 0.16b	1.68 ± 0.52bc
	N	26.12 ± 1.30a	22.30 ± 2.14a	9.91 ± 2.18a	5.44 ± 2.87b
	NP	17.06 ± 2.27b	5.38 ± 1.22b	11.85 ± 2.09a	16.67 ± 3.13a
AN (g m^−2^)	CK	2.71 ± 0.10b	1.91 ± 0.18ab	0.80 ± 0.06	0.57 ± 0.13b
	P	3.64 ± 0.15a	1.62 ± 0.32b	0.77 ± 0.14	0.31 ± 0.12b
	N	1.88 ± 0.36c	2.44 ± 0.74a	0.54 ± 0.15	1.07 ± 0.07a
	NP	3.64 ± 0.51a	1.51 ± 0.07b	0.72 ± 0.17	1.05 ± 0.32a
AP (g m^−2^)	CK	1.47 ± 0.27b	1.31 ± 0.09c	1.62 ± 0.08b	1.36 ± 0.08b
	P	2.68 ± 0.20a	1.56 ± 0.12b	1.80 ± 0.06b	1.87 ± 0.15a
	N	2.44 ± 0.24a	1.58 ± 0.03b	1.77 ± 0.09b	1.55 ± 0.17ab
	NP	2.99 ± 0.50a	1.92 ± 0.18a	2.62 ± 0.29a	1.65 ± 0.25ab

### Relationship of C, N, and P content and ecological stoichiometry in the shoot-soil system

Shoot C was significantly positively correlated with soil P, but significantly negatively correlated with soil C:P, N:P, and NN (Table 4). With the exceptions of soil N and AP, shoot N was significantly correlated with soil nutrient and stoichiometry, which was negatively correlated with soil C, P, and C:N, but positively correlated with soil C:P, N:P, NN, and AN. Shoot P had no correlation with soil nutrients and stoichiometries. As opposed to shoot N, shoot C:N positively correlated with soil C, N, P, and C:N, but negatively correlated with soil C:P, N:P, NN, AN, and AP. Shoot C:P and N:P had minimal correlations with soil nutrient and stoichiometries; e.g., only shoot N:P showed a significant positive correlation with soil AN and AP.

**Table 4:** Correlation (*R* value) between shoot and soil nutrient contents and stoichiometries in alfalfa grassland.

Index	Soil C	Soil N	Soil P	Soil C:N	Soil C:P	Soil N:P	Soil NN	Soil AN	Soil AP
Shoot C	0.324NS	0.345NS	0.510*	0.101NS	− 0.548**	− 0.469*	− 0.411**	− 0.213NS	− 0.033NS
Shoot N	− 0.726***	− 0.396NS	− 0.764***	− 0.531**	0.712***	0.793***	0.404**	0.354*	0.195NS
Shoot P	− 0.003NS	0.157NS	− 0.194NS	− 0.091NS	0.350NS	0.385NS	0.022NS	− 0.227NS	− 0.145NS
Shoot C:N	0.676***	0.439*	0.774***	0.434*	− 0.754***	− 0.777***	− 0.494***	− 0.382**	− 0.214NS
Shoot C:P	− 0.059NS	− 0.065NS	0.154NS	− 0.051NS	− 0.328NS	− 0.306NS	− 0.143NS	0.273NS	0.261NS
Shoot N:P	− 0.326NS	− 0.251NS	− 0.153NS	− 0.215NS	− 0.037NS	− 0.007NS	0.101NS	0.458**	0.324*

The linear regression was analyzed with the model y = ax + b. The linear regression between shoot and soil C, N, P, C:N, C:P, N:P were analyzed with the data in the first cut of 2014 and second cut of 2015 (*n* = 24). The linear regression between shoot C, N, P, C:N, C:P, N:P and soil NN, AN, AP contents were analyzed with the data in the first cut of 2014, first and second cuts of 2015 and first cut of 2016 (*n* = 48).

*P < 0.05; **P < 0.01; ***P < 0.001.

*NS* no correlation.

### Integrated responses of soil-shoot-biomass systems under N and P fertilization

**Figure 2:**
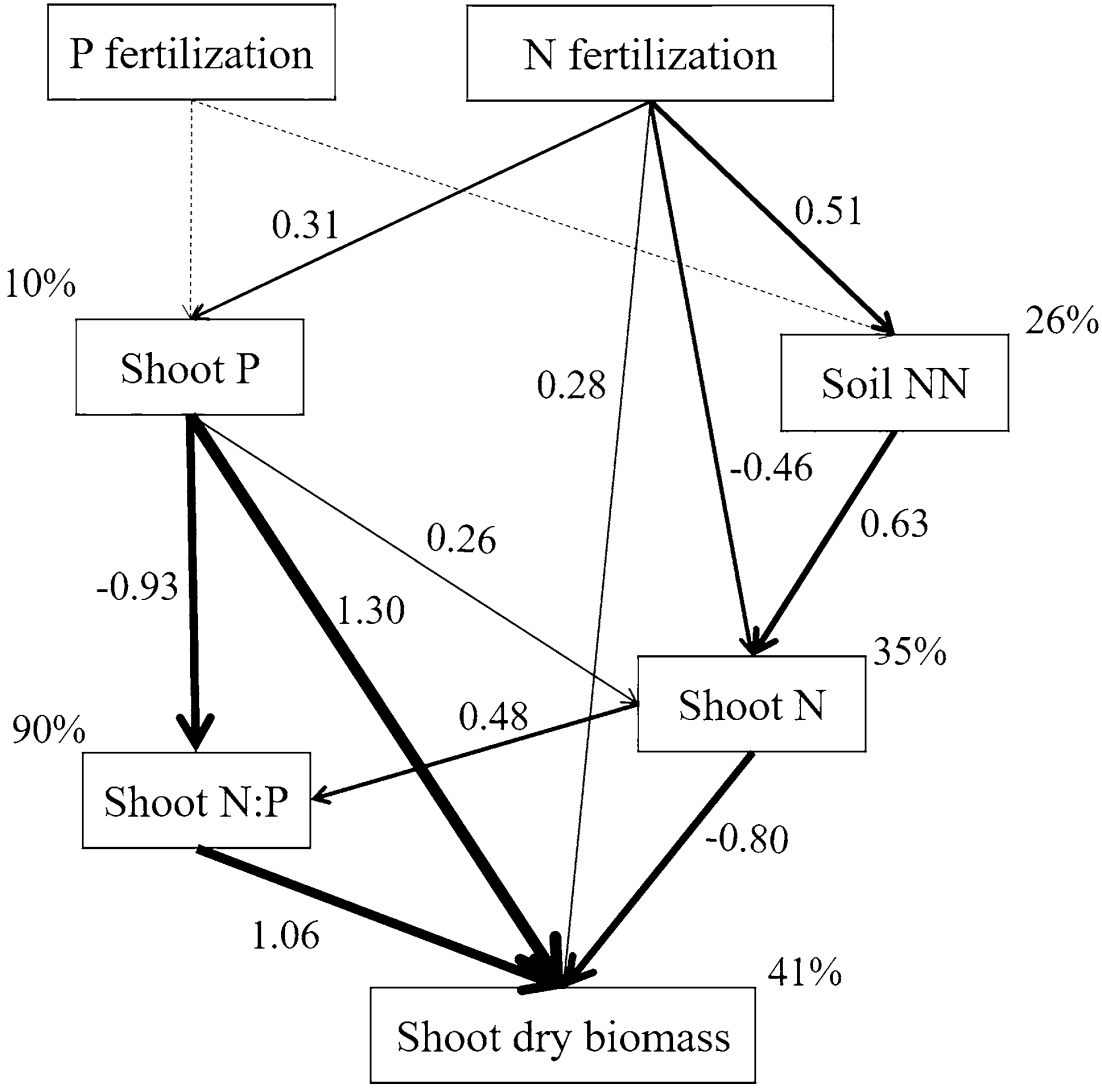
Structural equation models for the paths of N and P fertilization affecting soil and shoot N and P nutrients, stoichiometry and shoot dry biomass. The final models fitted the data well: χ^2^ = 7.442, df = 4, χ^2/^df = 1.861, *P* = 0.114, GFI = 0.954, RMSEA = 0.135; the solid arrows indicate significant positive relations (*P* < 0.05), and the dashed arrow indicates no relation. Numbers near the arrows are the standardized path coefficients and the width of the arrows is proportional to the strength of the relationship. The percentages near the endogenous variables indicate the variance explained by the model (*R*^2^).

Using a structural equation modeling (SEM) approach, the integrated responses of soil-shoot system nutrients and stoichiometry after N and/or P fertilization were elucidated (Fig. 2). Based on the SEM model, the path of P fertilization effects the soil-shoot system was not pass. Therefore, we deleted the SEM model analysis of P fertilizer. Based on the effect of N fertilization, including all treatments, the SEM model explained 90% of the variation in shoot N:P, 41% of the variation in biomass, 35% of the variation in shoot N, 26% of the variation in soil NN, and 10% of the variation in shoot P. Specifically, N fertilization exerted positive direct effect on soil NN, which positively affected shoot N. N fertilization showed a positive direct effect on shoot P that greatly reduced shoot N:P. N fertilization negatively affected shoot N. Shoot N showed a positive effect on shoot N:P. Shoot P and shoot N:P showed a strong positive, but shoot N showed a negative effect on shoot dry biomass. Taken together, N fertilization exerted a positive total effect on shoot P but showed a complex effect on shoot N, which induce a negative effect on shoot N:P. The decrease of shoot N:P induced by N fertilizer promoted the increase of alfalfa biomass.

## Discussion

### Characteristics of alfalfa C, N, and P concentration and stoichiometry under fertilization

Nutrient stoichiometry could be used as a tool for analyzing the balance between the nutrition elements required by organisms and affecting nutrient cycling^12^. C:N and C:P represent the ability of plants to assimilate C and the efficiency of plant C sequestration, that is, the storage capacity and accumulation rate of C, while N:P can reflect the restriction of plant growth by N or P^25,26^. In our study, shoot C and C:N were higher but shoot N was lower under fertilization treatment, and this was significant under P fertilization. This is mainly because the increase in biomass caused by fertilization dilutes the shoot N concentration^27^ (Fig. 2 and Figure S1), creating a negative correlation between C and N in the element stoichiometry of higher terrestrial plants^12^, increasing C:N. In particular, P fertilization resulted in the relative deficiency of N available for absorption by alfalfa in the soil (Table 3), and young alfalfa leading to the weakening of biological nitrogen fixation ability^21^, subsequent shoot N concentration decrease, and C:N increase.

P fertilization increased the biomass of alfalfa, but only about 20% of P fertilization was absorbed by plants in the first fertilization year^21^. Shoot P concentration decreased but C:P increased at the first year. The biomass significantly higher under N and NP fertilization mainly due to that N application accelerates soil phosphatases activities; therefore, soil organic P is mineralized to a plant-available status at a faster rate, which is subsequently taken up with greater efficiency by the plant^8,28^. The plant P concentration was higher under N and NP fertilization at the first cut of each year, which correspondingly lowered C:P. Further, because soil P deficiency is a serious problem in the Loess Plateau^21,29^, the effect of N addition alone was not as good as NP fertilization. However, at the second cut in 2015, N and NP fertilization decreased shoot P concentration. This is because appropriate clipping can enhance photosynthesis and promote rapid growth recovery of plants by increasing the distribution of N to shoots and providing resources for the regeneration of shoots^30^. In the second cut of 2015, the biomass significantly higher under N and NP fertilization (Figure S1), but annual addition of N fertilizer (April) reduced the soil N (August) due to cutting and growth, which reduced conversion rate of available P, resulting in limited uptake of P and the dilution of P in shoot and therefore higher C:P. In this study, shoot P concentration did not differ between P fertilizer and CK in 3 years; even shoot P concentration was lower after P fertilizer treatment than in the CK condition in 2016, indicating that a one-time application of P fertilizer may play a role for less than 3 years.

N addition accelerate P cycles and restrains the nitrogen fixation ability, meaning plants absorb more P than N^31^. N:P under N and NP fertilization was significantly lower than in the CK condition at the first cut of each year, and N and NP fertilization negatively affected N:P, indicating that N and NP fertilization can alleviate the phosphorus limitation of alfalfa. At the second cut of 2015, N and NP fertilization increased shoot N:P. The compensatory growth of alfalfa after cutting resulted in redistribution of storage compounds and an increase in the allocation of newly absorbed N to shoots^32^. In our study, the variation of shoot N:P was mainly caused by the changes in shoot P (Fig.2), consistent with the argument that P responses after fertilization are more variable and largely drive the observed changes in N:P values^10^.

Cui et al.^8^ found that grass tended to have higher C:N and C:P than other plant functional groups. In our study, the mean plant C:N and C:P were 16.52 and 469.24, consistent with the previous study. In different ecosystems, N:P represents the limit of nitrogen or phosphorus. A review of 40 fertilization studies showed that N:P > 16 indicated a P limitation, whereas N:P < 14 indicated a N limitation, and plant growth was co-limited by N and P together when N:P fell between 14 and 16^33^. However, some studies have found that communities were N limited when N:P was < 21, whereas N:P > 23 might indicate a P limitation^26^. To some extent, fertilization breaks the inherent nutrient state of soil and alleviates the plant’s demand for restrictive elements. In this study, all samples exceeded the limit threshold of P reported by previous studies^26,33^, and N and NP fertilization decreased shoot N:P and increased alfalfa biomass (Fig. S1), indicating that P is severely restricted in the Loess Plateau, and N fertilizer can alleviate this situation while increasing yield. Further, NP fertilization can not only directly increase the content of P (mobile and non-mobile P) in the soil, but also activate more inorganic P through N-stimulated phosphatase, which significantly increased shoot P content and alfalfa biomass (Table S5 and Figure S1), indicating that the addition of combined N and P has a more obvious mitigation effect than application of a single N supplement.

### Characteristics of soil C, N, and P content and stoichiometry under fertilization

Soil nutrient stoichiometry patterns could enhance our understanding of nutrient cycling and biological processes in terrestrial ecosystems^34,35^, creating a useful indicator for evaluating soil management. Suo et al.^36^ found that soil stoichiometry exhibited smaller variations than plant tissue. Inconsistently, the present study found soil C:N significantly higher under N fertilization than CK at the first cut of 2014. This is mainly because the application of N fertilizer to the soil can lead to the build-up of soil organic matter and therefore increase soil organic carbon^37^. Similarly, soil C:P is significantly higher under N and NP fertilizers than CK at the first cut because there is higher soil C. Soil P content did not change under N and NP fertilization in the first year, probably because there is very little P in the soil. The total P may also mask the slight variation in available P, as shown by the significantly higher soil AP under fertilization (Table 3 and Fig. 2). Fertilization significantly decreased soil C:P and N:P at the second cut of 2015, because P content significantly increased after the application of fertilizer (Table 3) and crop residues recycle in the soil with a positive P budget^38^. In addition, the higher biomass induced by N and NP fertilization would account for the fact that plant-available P was markedly lower, which would have stimulated the production of phosphatase enzymes by soil bacteria, and in turn resulted in the mineralization of soil organic P^39,40^.

In the present study, the mean soil C:N, C:P, and N:P ratios were 8.11, 17.40, and 1.33, respectively, lower than the previously reported mean C:N:P ratio in Chinese soil of 134:9:1^34^. This is mainly due to soil stoichiometry having a high spatial heterogeneity and being affected by local abiotic and aboveground biotic factors^34,36^. Soil erosion in the Loess Plateau aggravates the loss of soil nutrients and severely decreases soil quality^1^. Additionally, in ecosystems with low levels of soil nutrients, available nutrients are tightly cycled between plants and organic matter rather than entering the soil pool^41^, further aggravating soil nutrient limitations.

### Correlation of shoot-soil C, N, and P content and stoichiometry

Plants and soil are closely linked and interact with each other in natural ecosystems, but few studies have reported how soil nutrients relate to the nutrients of plants^14^. In our study, soil P was significantly correlated with shoot C, N, and C:N, which means that the growth of alfalfa is greatly affected by soil P and that differences in soil P content are a major driver of variation in elemental stoichiometry^42^. Soil P had no significant effect on shoot P, mainly because the one-time application of P fertilizer only played a significant role in 2014 and the first cut of 2015 (Supplementary Tables S1–S4). Fertilization supplemented the deficiency of soil P in 2014, but a substantial part of P accumulates in the soil as residual P rather than being absorbed by the plant^21^. Later growth of alfalfa is limited by low rainfall, immobile P and the complexity of P and N coabsorption. Studies have shown that N and P are in close stoichiometric balance in most ecosystems^43^ and therefore are not independent of each other^44^, which may be inconsistent with our results showing that soil N had no effect on shoot nutrients and stoichiometry. Although N and P are two of the more limiting nutrients to plant growth, they differ profoundly in their bioavailability in the soil, with N being relatively mobile, and P being relatively immobile and often very patchily distributed both spatially and temporally^10^. Additionally, legumes are relatively abundant in N sources because of their biological nitrogen fixation ability. Soil N does not appear to be the only determinant of plant nutrients^31^. In the present study, shoot N and C:N were easily influenced by soil nutrient and stoichiometry, which indicated that variation of soil nutrients caused by fertilization had a consistent influence on N absorption by alfalfa at different growth stages.

## Conclusions

N and NP fertilizer can alleviate the P limitation of alfalfa. The effect was more obvious under the application of combined N and P than under N supplementation alone. P responses after fertilization were more variable and largely drove the observed changes in the C:P and N:P ratios.

## Materials and methods

### Study site description

The field research was conducted at the Loess Plateau Experimental Station of Lanzhou University, Gansu Province, China (35°40′N, 107°51′E, 1298 m a.s.l.). There is a typical continental climate in this region. The annual mean temperature is 8–10 °C and annual total solar irradiation duration is 2300–2700 h. Annual average precipitation is 563 mm and 70% of the rain falls from July to September. The soil is Heilu soil (Entisol of FAO classification), which is a sandy loam with 7% sand, 70% silt and 23% clay, representative of the major soil in this area. A landrace of alfalfa (*M. sativa* cv. Longdong) is grown in the station and the preceding crops at the experiment site were cereal crops such as maize (*Zea mays* L.) or winter wheat (*Triticum aestivum* L.).

### Experimental design

The study was conducted from April 2014 to July 2016. Alfalfa was sown at the seeding rate 22.5 kg ha^−1^ at April 2014. In each plot of 3 m × 4 m in size, there were 10 lines of plants with line-space 30 cm. We set no fertilization (CK), only N fertilization (N, 100 kg N ha^−1^, added as urea, N ≥ 46%), only P fertilization (P, 120 kg P_2_O_5_ ha^−1^, added as calcium superphosphate, P_2_O_5_ ≥ 16%) and N and P combined fertilization (NP, 100 kg N ha^−1^, 120 kg P_2_O_5_ ha^−1^) treatments. There were 3 repetitions for each treatment with a total 12 plots and 30-cm interval was set between two plots. The experiment adopted a completely random block design. The N fertilizer was applied before sowing at 2014 and mid-April at 2015 and 2016. The P fertilizer one-time applied before sowing (Table 5).

**Table 5:** Fertilization time.

	2014	2015	2016
N fertilizer	April (before sowing)	Mid-April	Mid-April
P fertilizer	One-time application	–	–

### Sampling and measurement

Shoots were sampled at the early flowering stage of the first to fourth cuts on August 2014, June 2015, August 2015, and June 2016. 0.5 m aboveground alfalfa of uniform growth were chosen as the shoot. Shoots were oven-dried at 105 °C for 10 min then oven-dried at 65 °C to constant weight. Dried samples were ground uniformly and passed through a 1.0 mm sieve for further measurement.

In each plot, soil samples from 0–10, 10–20, 20–30 and 30–60 cm depths were collected using soil-drilling methods at the same time as shoot collection. Soil samples were air-dried, roots and stones removed, and passed through 2 mm and 0.25 mm sieves for further measurement. Soil bulk density samples were obtained randomly from five points before sowing by volumetric rings.

Because the total nutrients in the soil are not easily changed, the C, N, and P were only detected in August 2014 and 2015, while NN, AN, and AP were detected after each cut. C concentration was determined using the K_2_Cr_2_O_7_–H_2_SO_4_ oxidation method. N concentration was measured using the automatic-Kjeldahl method with a Kjeldahl auto-analyzer (FOSS 8400, Shanghai, China). P concentration was extracted using molybdenum antimony colorimetric method with a spectrophotometer (UV-2102 PCS, Shanghai, China). Soil ammonium nitrogen (NH_4_^+^, AN) was extracted in 2 mol L^−1^ KCl and determined using an indophenol blue colorimetric method (UV-2102 PCS, Shanghai, China). Soil nitrate nitrogen (NO_3_^−^, NN) was extracted in 2 mol L^−1^ KCl and measured using an ultraviolet spectrophotometry method (UV-2102 PCS, Shanghai, China). Soil available phosphorus (AP) was extracted in 0.5 mol L^−1^ sodium bicarbonate (NaHCO_3_) and determined using the Olsen method. The soil bulk density was determined using the soil core method and obtained by calculating the ratio of soil mass to total volume (g cm^−3^) after oven dried at 105 °C to a constant weight^45^.

### Statistics and analysis

Soil C, N, P, NN, AN, and AP content was calculated by the following formula:

$${\text{SNC}} = \frac{{\sum\nolimits_{{{\text{i}} = 1}}^{{\text{n}}} {\uprho _{{\text{i}}} \times {\text{C}}_{{\text{i}}} \times {\text{T}}_{{\text{i}}} } }}{100}$$

In the formula, SNC is the soil C, N, P content (kg m^−2^) or the soil NN, AN, AP content (g m^−2^), ρ_i_ indicates the bulk density (g cm^−3^) at the i layer, C_i_ indicates the C, N, P concentration (g kg^−1^) or the NN, AN, AP concentration (mg kg^−1^) at the i layer, T_i_ indicates the thickness (cm) of the soil at the i layer, n indicates the number of layer.

Excel 2010 (Microsoft, Redmond, WA, USA) was used for data handling and plotting. All analyses were conducted with SPSS software (v. 21.0; IBM SPSS, Armonk, NY, USA). Duncan’s test of the one-way analysis of variance (ANOVA) was performed to assess the effects of fertilizer on the shoot-soil nutrients (C, N, P, NN, AN, and AP) and ratio (C:N, C:P, or N:P). Linear regression was performed to assess the relationship between shoot nutrient or stoichiometry and soil nutrient or stoichiometry.

Structural equation modeling (SEM) was performed using the AMOS software (IBM SPSS AMOS 25, Chicago, IL, USA) to examine the direct and indirect effects of N and/or P fertilization on nutrients and stoichiometry of soil and shoot, and shoot dry biomass. The data of soil and shoot nutrients and stoichiometry in SEM are their original values of each plot at each cut. The underlying assumption of SEM was that N and P fertilization could directly affect the nutrients of soil and shoot, and could also indirectly affect shoot nutrients by affecting soil nutrients, which further had indirect effects on shoot stoichiometry and dry biomass. Next, we examined the modification indices to ensure that no important paths were left out of the model, and then we removed paths with coefficients that were not significant at *P* < 0.05. The adequacy of the models was determined with a chi-squared (χ^2^) test, the goodness of fit index (GFI) and root square mean errors of approximation (RMEA). Adequate model fits are indicated by χ^2^ test, large GFI and small RMSEA, which suggest that there is a small difference between the modeled and observed values. The coefficient of each causal relation was expressed as a standardized path coefficient, which can represent the magnitude of the direct effect of the predictor on the outcome. The standardized total effect (direct plus indirect effects) stands for the ultimate contributions of one variable to another.

Plant studies were carried out in accordance with relevant institutional, national or international guidelines.

## Supplementary Information

Supplementary Information.
