# Supplementary material for: Shoot-soil ecological stoichiometry of alfalfa under nitrogen and phosphorus fertilization in the Loess Plateau
Source: Sci Rep. 2021 Jul 22;11:15049. doi: 10.1038/s41598-021-94472-2 (PMC8298445; doi:10.1038/s41598-021-94472-2)
Supplement: Supplementary file 1 — Supplementary Information. [file 41598_2021_94472_MOESM1_ESM.doc]

# **Shoot-soil ecological stoichiometry of alfalfa under nitrogen and phosphorus fertilization in the Loess Plateau**

Jiaoyun Lu^1^, Hong Tian^1^, Heshan Zhang^1^, Junbo Xiong^1^, Huimin Yang^2*^, Yang Liu^1*^

^1^ Key Laboratory of Animal Embryo Engineering and Molecular Breeding of Hubei Province, Institute of Animal Husbandry and Veterinary, Hubei Academy of Agricultural Science, Wuhan 430064, China

^2^ State Key Laboratory of Grassland Agro-ecosystems; College of Pastoral Agriculture Science and Technology, Lanzhou University, Lanzhou 730020, China

Corresponding Author:

Huimin Yang^2*^

State Key Laboratory of Grassland Agro-ecosystems, College of Pastoral Agriculture Science and Technology, Lanzhou University, 768 Jiayuguanxi Road, Lanzhou 730020, China.

Email address: huimyang@lzu.edu.cm

Yang Liu^1*^

Key Laboratory of Animal Embryo Engineering and Molecular Breeding of Hubei Province, Institute of Animal Husbandry and Veterinary, Hubei Academy of Agricultural Science, Yaoyuan 1, Hongshan, Wuhan, 430064, China

Email address: liuyang430209@163.com

**Supplementary Information**

**Table S1** Correlation (*R* value) between shoot and soil nutrient contents and stoichiometries in 2014

| Index | Soil C | Soil N | Soil P | Soil C:N | Soil C:P | Soil N:P | Soil NN | Soil AN | Soil AP |
| --- | --- | --- | --- | --- | --- | --- | --- | --- | --- |
| Shoot C | -0.210NS | 0.504NS | 0.344NS | -0.453NS | -0.408NS | 0.072NS | -0.636* | 0.654* | 0.449NS |
| Shoot N | -0.305NS | 0.129NS | -0.429NS | -0.326NS | 0.040NS | 0.392NS | 0.506NS | -0.294NS | -0.497NS |
| Shoot P | 0.096NS | 0.432NS | -0.756** | -0.140NS | 0.671* | 0.832*** | 0.519NS | -0.094NS | 0.156NS |
| Shoot C:N | 0.077NS | 0.166NS | 0.500NS | -0.028NS | -0.279NS | -0.256NS | -0.710** | 0.566NS | 0.569NS |
| Shoot C:P | -0.108NS | -0.173NS | 0.842*** | -0.017NS | -0.731** | -0.732** | -0.749** | 0.338NS | 0.065NS |
| Shoot N:P | -0.140NS | -0.231NS | 0.814*** | -0.014NS | -0.745** | -0.750** | -0.671* | 0.262NS | -0.055NS |

Note: The linear regression was analyzed with the model y=ax+b (*n*=12). *: *P*＜0.05; **: *P*＜0.01; ***: *P*＜0.001; NS: no correlation. The same below.

**Table S2** Correlation (*R* value) between shoot and soil nutrient contents in the first cut of 2015

| Index | Soil NN | Soil AN | Soil AP |
| --- | --- | --- | --- |
| Shoot C | -0.526NS | -0.304NS | -0.232NS |
| Shoot N | 0.238NS | 0.274NS | -0.025NS |
| Shoot P | -0.100NS | -0.471NS | 0.833*** |
| Shoot C:N | -0.481NS | -0.342NS | -0.172NS |
| Shoot C:P | -0.259NS | 0.236NS | -0.842*** |
| Shoot N:P | 0.193NS | 0.517NS | -0.635* |

**Table S3** Correlation (*R* value) between shoot and soil nutrient contents and stoichiometries in the second cut of 2015

| Index | Soil C | Soil N | Soil P | Soil C:N | Soil C:P | Soil N:P | Soil NN | Soil AN | Soil AP |
| --- | --- | --- | --- | --- | --- | --- | --- | --- | --- |
| Shoot C | 0.045NS | -0.152NS | 0.138NS | 0.319NS | -0.124NS | -0.144NS | -0.392NS | -0.362NS | -0.506NS |
| Shoot N | -0.552NS | -0.330NS | -0.451NS | -0.385NS | 0.461NS | 0.509NS | -0.264NS | 0.069NS | -0.390NS |
| Shoot P | 0.043NS | -0.189NS | -0.259NS | 0.433NS | 0.290NS | 0.240NS | -0.703* | 0.424NS | -0571NS |
| Shoot C:N | 0.502NS | 0.195NS | 0.413NS | 0.522NS | -0.413NS | -0.469NS | -0.028NS | -0.212NS | 0.032NS |
| Shoot C:P | -0.074NS | 0.126NS | 0.343NS | -0.387NS | -0.377NS | -0.324NS | 0.616* | -0.537NS | 0.529NS |
| Shoot N:P | -0.312NS | 0.029NS | 0.125NS | -0.623* | -0.158NS | -0.081NS | 0.609* | -0.404NS | 0.490NS |

**Table S4** Correlation (*R* value) between shoot and soil nutrient contents in the first cut of 2016

| Index | Soil NN | Soil AN | Soil AP |
| --- | --- | --- | --- |
| Shoot C | 0.105NS | 0.082NS | -0.280NS |
| Shoot N | 0.061NS | -0.154NS | -0.122NS |
| Shoot P | 0.322NS | 0.343NS | -0.070NS |
| Shoot C:N | -0.062NS | 0.132NS | 0.008NS |
| Shoot C:P | -0.314NS | -0.392NS | -0.010NS |
| Shoot N:P | 0.501NS | 0.189NS | -0.051NS |

**Table S5** Characteristics of alfalfa C, N, and P content under N and P fertilization

| Nutrient content | Treatment | Sample time | | | |
| --- | --- | --- | --- | --- | --- |
|  |  | 2014-1st | 2015-1st | 2015-2nd | 2016-1st |
| C  (kg ha^-1^) | CK | 1393.11±55.18c | 1137.54±55.05d | 1015.46±25.43b | 1564.73±466.72b |
|  | P | 1879.20±142.05a | 1981.84±67.99b | 1184.95±152.59b | 3318.32±556.02a |
|  | N | 1643.49±138.65b | 1640.52±35.64c | 1340.47±58.78b | 2531.85±389.33a |
|  | NP | 2042.60±63.17a | 3184.38±310.11a | 2816.72±293.29a | 3295.28±568.18a |
| N  (kg ha^-1^) | CK | 102.63±10.75c | 69.65±13.12c | 63.42±2.75b | 97.23±16.97c |
|  | P | 120.69±4.42b | 103.60±0.29b | 65.05±9.28b | 189.72±12.98ab |
|  | N | 115.41±7.54bc | 108.73±5.56b | 76.28±5.70b | 132.97±20.96bc |
|  | NP | 136.73±6.88a | 186.11±7.71a | 164.97±15.04a | 211.37±67.20a |
| P  (kg ha^-1^) | CK | 3.01±0.36c | 2.23±0.20c | 2.13±0.10b | 3.80±0.71b |
|  | P | 2.33±0.16d | 3.89±0.14b | 2.56±0.29b | 8.18±1.34a |
|  | N | 3.77±0.29b | 3.65±0.32b | 2.42±0.12b | 7.38±1.27a |
|  | NP | 5.76±0.39a | 8.31±0.60a | 4.97±1.09a | 9.72±2.99a |

Note: Calculate the content value by multiplying the concentration by the biomass. Values are presented as mean±SD (*n*=3). Different lowercase letters indicate significant differences (*P*<0.05) between different treatment, no letters indicate no significant difference between treatments. C: organic carbon; N: total nitrogen; P: total phosphorus.

**Figure Captions**

**
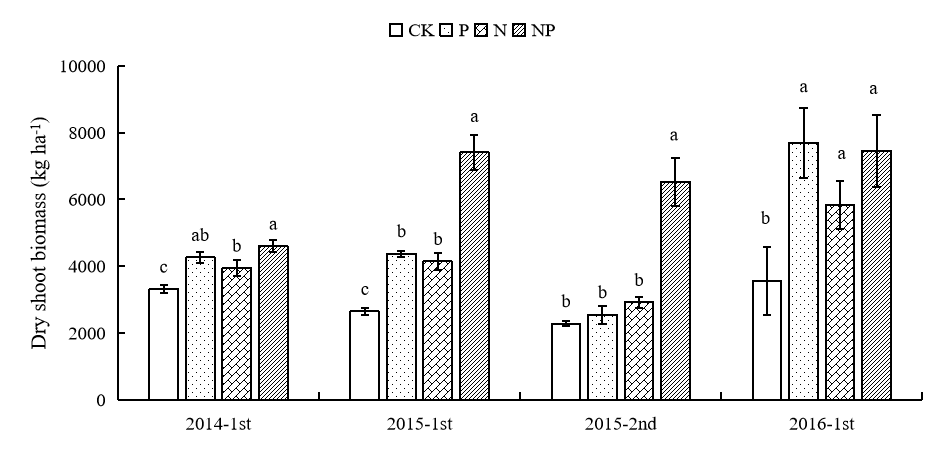
**

**Figure S1** Dry shoot biomass of alfalfa under N and P fertilization

Different lowercase letters on the bars mean significant difference under fertilization in the same cut (*P*<0.05). Data showed by mean±SD (*n*=3).
